# Global trends and prospects about synaptic plasticity in Alzheimer’s disease: a bibliometric analysis

**DOI:** 10.3389/fnagi.2023.1234719

**Published:** 2023-09-05

**Authors:** Yingying Zhang, Junyao Zhang, Yinuo Wang, Junyan Yao

**Affiliations:** ^1^Department of Anesthesiology, Shanghai General Hospital, Shanghai Jiao Tong University School of Medicine, Shanghai, China; ^2^Department of Anesthesiology, Shanghai East Hospital, Tongji University School of Medicine, Shanghai, China

**Keywords:** Alzheimer’s disease, synaptic plasticity, bibliometric analysis, hotspots, VOSviewer, CiteSpace, co-citation

## Abstract

**Background and purpose:**

In recent years, synaptic plasticity disorders have been identified as one of the key pathogenic factors and the early pathological characteristics of Alzheimer’s disease (AD). In this study, we tried to use bibliometric analysis to gain a systematic understanding about synaptic plasticity in Alzheimer’s disease.

**Methods:**

We extracted relevant publications from the Web of Science Core Collection (WoSCC) on August 29th, 2022. Then, we used CiteSpace, VOSviewer and other online bibliometric platforms^1^ to further analyze the obtained data.

**Results:**

A total of 2,348 published articles and reviews about synaptic plasticity in AD from 2002 to 2022 were identified. During the past two decades, the overall trends of the numbers and citations of manuscripts were on the rise. The United States was the leading country with the largest number of publications which showed its crucial role in this field. The collaboration network analysis showed that the United States and China had the most frequent collaboration. In addition, Harvard University was the institution with the greatest number of publications and cited times. Among all authors, Selkoe DJ was the most influential author with the greatest cited times. The journal of Alzheimer’s disease published the maximum number of documents in the field of synaptic plasticity in AD within 20 years. Furthermore, the results of keywords burst detection showed that the hot topics have shifted from the synaptic transmission, precursor protein and plaque formation to neuroinflammation, microglia and alpha synuclein.

**Conclusion:**

This study analyzed 2,348 publications with 82,025 references covering the topic of synaptic plasticity in AD and presented the research trends. The results indicated that neuroinflammation, microglia and alpha synuclein were the current research hotspots, which implied the potential clinical applications to AD.

## Introduction

It has been forecasted that by 2050, the prevalence of dementia will double in Europe and triple worldwide (Scheltens et al., 2021). Alzheimer’s disease (AD), a major cause of dementia, has becoming a global health concern. Nowadays, with the escalation of the aging process, the incidence of AD is also on increasing. The prevalence of AD not only damages the elderly’s health, but also exerts a heavy burden on the family and society. Consequently, it is critical to find early diagnosis methods and effective therapeutic interventions for AD patients. Although the classical hypothesis of AD is the formation of amyloid plaques and neurofibrillary tangles (NFTs) mainly composed of amyloid-β (Aβ) peptides and hyper phosphorylated tau, the clinical therapeutic strategies based on this hypothesis still fail to achieve satisfactory results (Naseri et al., 2019). Thus, it is necessary to find new approaches to improve the treatment and diagnosis of AD.

The degeneration of synapses and dendritic spines are prior to the loss of neurons in many neurodegenerative diseases, especially in AD (Yu and Lu, 2012). As reported, the strength and efficiency of synaptic connections can be affected by the changes in the environment or the experience of the individuals (Krzeptowski et al., 2018; Mercerón-Martínez et al., 2021). This characteristic is defined as synaptic plasticity which is directly related to memory and learning process.

The mechanisms of synaptic plasticity refer to not only its morphological modifications, such as the regeneration of axons and the formation of new synapse but also its molecular changes that alter the cellular response to neurotrasmitters (Mercerón-Martínez et al., 2021). Over the years, long-term potentiation (LTP) and long-term depression (LTD), have generally been proposed to be the basis of cellular processes underlying learning and memory (Sweatt, 2016; Krzeptowski et al., 2018). LTP contributes to the formation of memory whereas LTD can inactive the memory (Nabavi et al., 2014). Therefore, LTP/LTD function together to modify the synaptic strength to encode the memories. Hence, when the efficacy of LTP decreases, the subjects’ cognitive capacity subsequently shows a declining trend. Studies from AD mouse models have also denoted that soluble oligomers of Aβ can inhibit the hippocampal LTP *in vivo* and afterwards adversely affect the synaptic transmission (Selkoe, 2002). Previous studies have demonstrated that learning and synaptic dysfunctions appear before the formation of amyloid plaques and NFTs, suggesting that the disorders of synaptic efficacy underlie the initial development of AD (Selkoe, 2002).

Bibliometric analysis is an important statistical method that is used to quantitatively analyze large amounts of heterogeneous publications (Chen et al., 2014). In recent years, bibliometric analysis has been used to provide clear insights into many scientific fields. CiteSpace is a Java web-based visualizing processing tool widely used in bibliometric analysis, which can offer good support in analyzing data from multiple perspectives, including different countries/regions, institutions, journals, authors, co-citation references and keywords (Chen, 2004).

Over the past two decades, many publications have illustrated the correlation between synaptic plasticity and AD, however, the systematic summaries of these studies are still insufficient. Hence, it is necessary to collect data from relevant publications to assist investigators in understanding the vast amount of literature on this subject. Through evaluating the existing data, we can help researchers to identify detailed research focus, global research trends, hot sports and guide future academic decisions in the field of synaptic plasticity in AD.

In this study, we aimed to provide a comprehensive understanding of the developments in the field of synaptic plasticity in AD by analyzing the data obtained from the WoSCC. Furthermore, we identified the research trends and potential hot spots which may be helpful in future research planning and decision-making.

## Methods

### Data source and search strategies

A literature search was conducted using the WoSCC database (Clarivate Analytics, Philadelphia, PA, United States) with the following search strategy: Topic = ((“synaptic plasticity” OR “synapse plasticity “) AND (“Alzheimer disease” OR “Alzheimer’s disease”)) AND language = English, limited time = from 2002 to 2022. We applied filters to limit the search to original articles and reviews, index = science citation index expanded (SCI-EXPANDED), timespan = 2002–2022. To reduce the bias incurred by frequent database updates, literature retrieval and data downloads were completed in 1 day on August 29th, 2022 by two authors (Yingying Zhang and Junyao Zhang) independently to obtain the primary data.

### Data extraction and collection

The manuscripts conducted by the aforementioned search strategies were then screened and recorded for titles, countries/regions, institutions, journals, authors and cited references. The WoSCC data were converted to txt format and imported into Microsoft Excel 2021, VOSviewer (version 1.6.18), CiteSpace (version 5.8.R3), online analysis platform of bibliometry (see Footnote 1) for further bibliometric analysis.

### Bibliometric analysis

All literature characteristics, including countries, institutions, journals, authors, co-citation references clusters and keywords with the strongest citation bursts were documented. The annual publication numbers and citation information of different countries/regions obtained from WoSCC were analyzed using Microsoft Excel 2021. CiteSpace v5.8.R3 was used to generate a visualization map in order to describe the co-cited authors/reference and to analyze keywords with strong citation bursts. The VOSviewer v1.6.18 was adopted to identify the intensity of cooperation between institutions and authors to illustrate the international influence of these institutions and authors in the field of synaptic plasticity in AD. In order to detect the research hotspots and predict research trends, we conducted the keywords co-occurrence network visualization map. Furthermore, the co-citation network visualization map of journals was also analyzed by VOSviewer.

## Results

### Bibliometric analysis of publication outputs and citation trend

**Figure 1:**
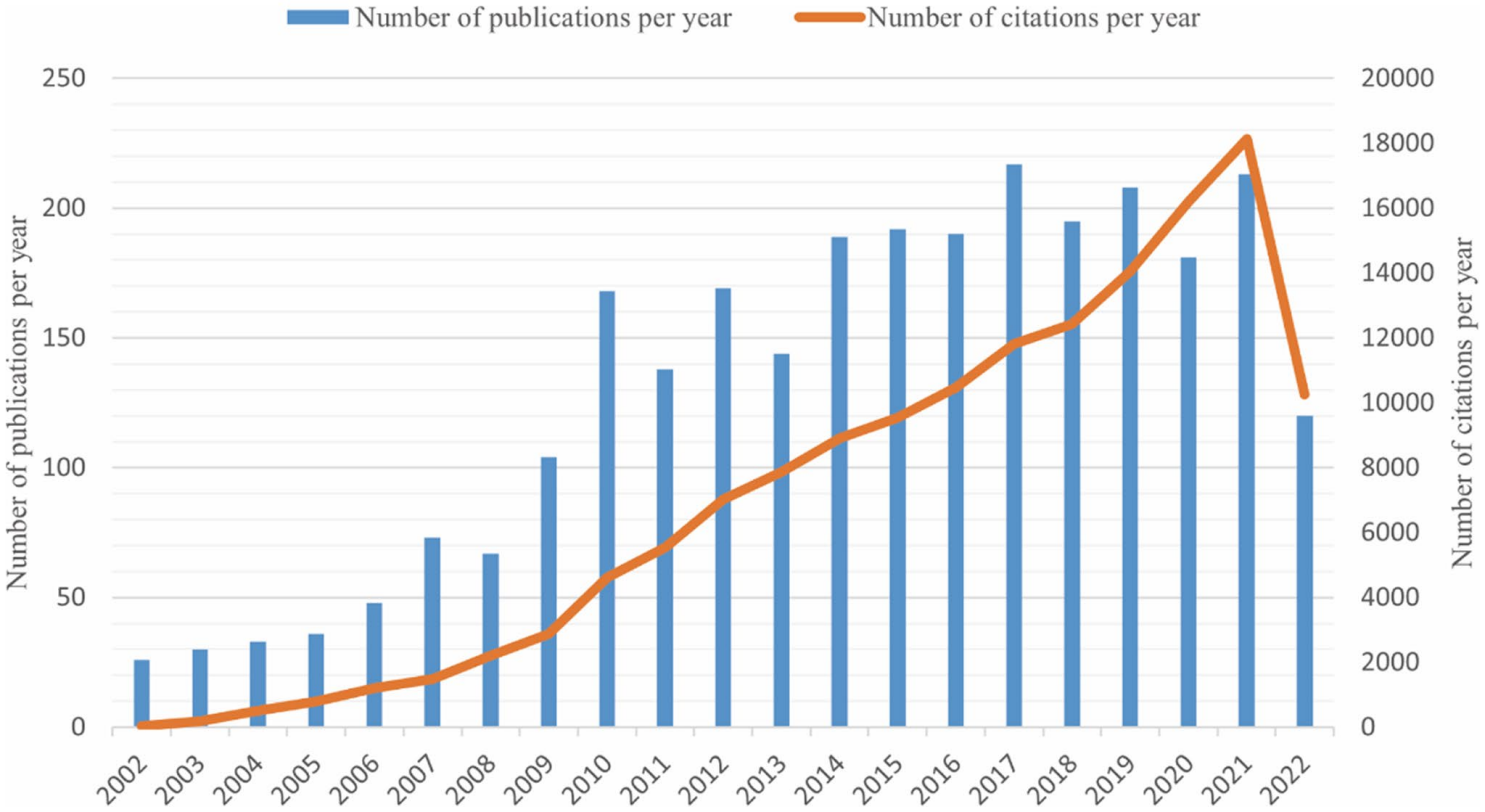
The trends of annual publications and citations on the topic of synaptic plasticity in Alzheimer’s disease from 2002 to 2022.

A total of 2,348 publications were included in our study. The number of publications and citations were concluded using Microsoft Excel 2021 (Figure 1). There were few articles before 2008, the overall trend was on the rise and reached its peak in 2017 with a total of 217 publications. This chart showed a surge increase in annual citation numbers between 2002 and 2021, presumably 62.87 times per item.

### Bibliometric analysis of countries and regions

**Figure 2:**
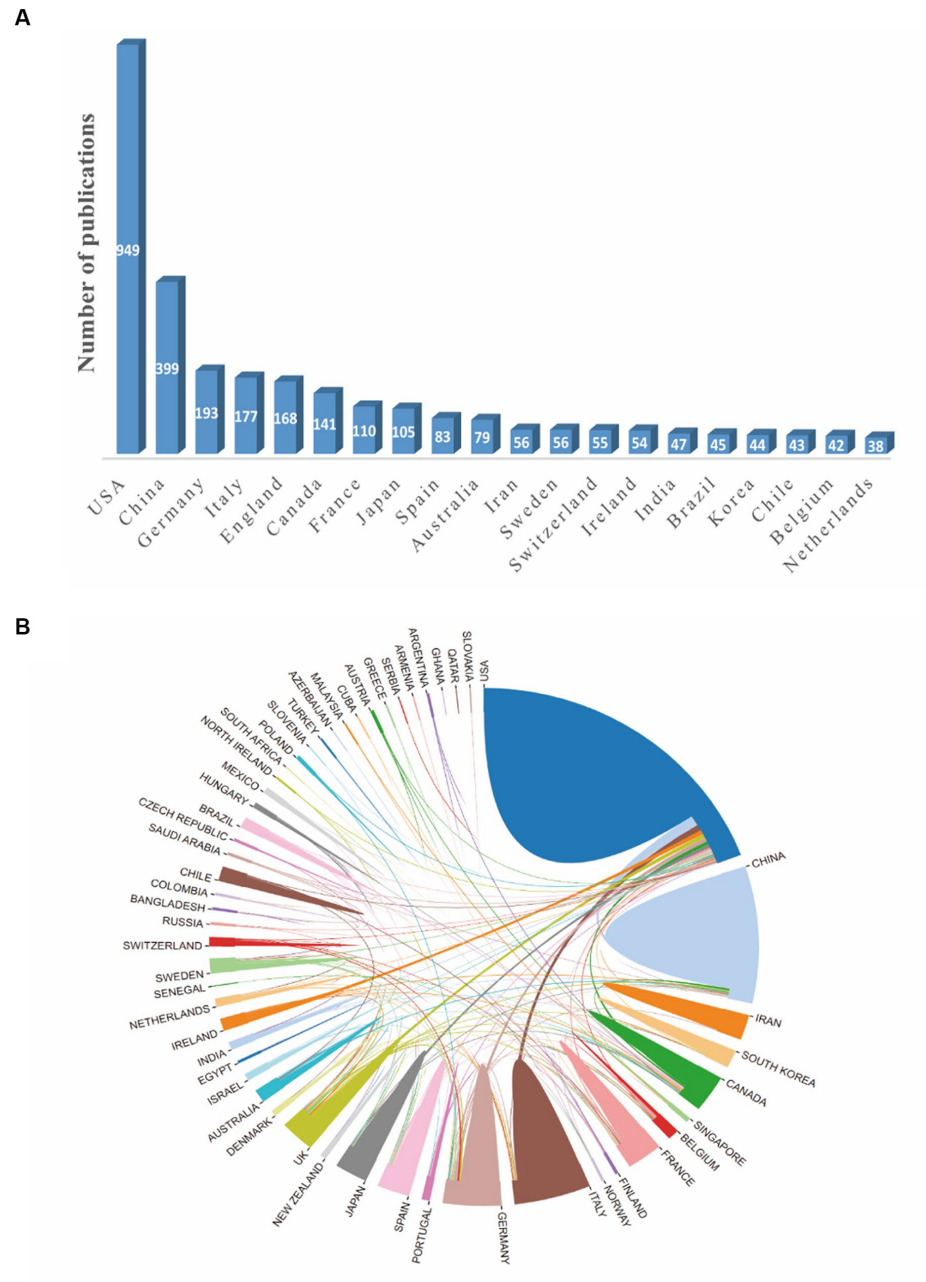
The network map of countries involved in the field of synaptic plasticity in Alzheimer’s disease. **(A)** The distribution map of the top 20 productive countries. **(B)** The visualization map of collaboration among countries generated by an online analytical platform.

The distribution map of the top 20 productive countries was shown in Figure 2A. The United States had the largest number of publications (949) in this field, followed by China (399), Germany (193) and Italy (177). A national cooperation network analysis was conducted by an online analytical platform (Figure 2B). In this map, the United States was the center of the national cooperation which showed frequently cooperation with China, Italy, Ireland, UK, Germany and Canada. Nevertheless, other countries displayed less international cooperation than the United States.

### Bibliometric analysis of institutions

**Figure 3:**
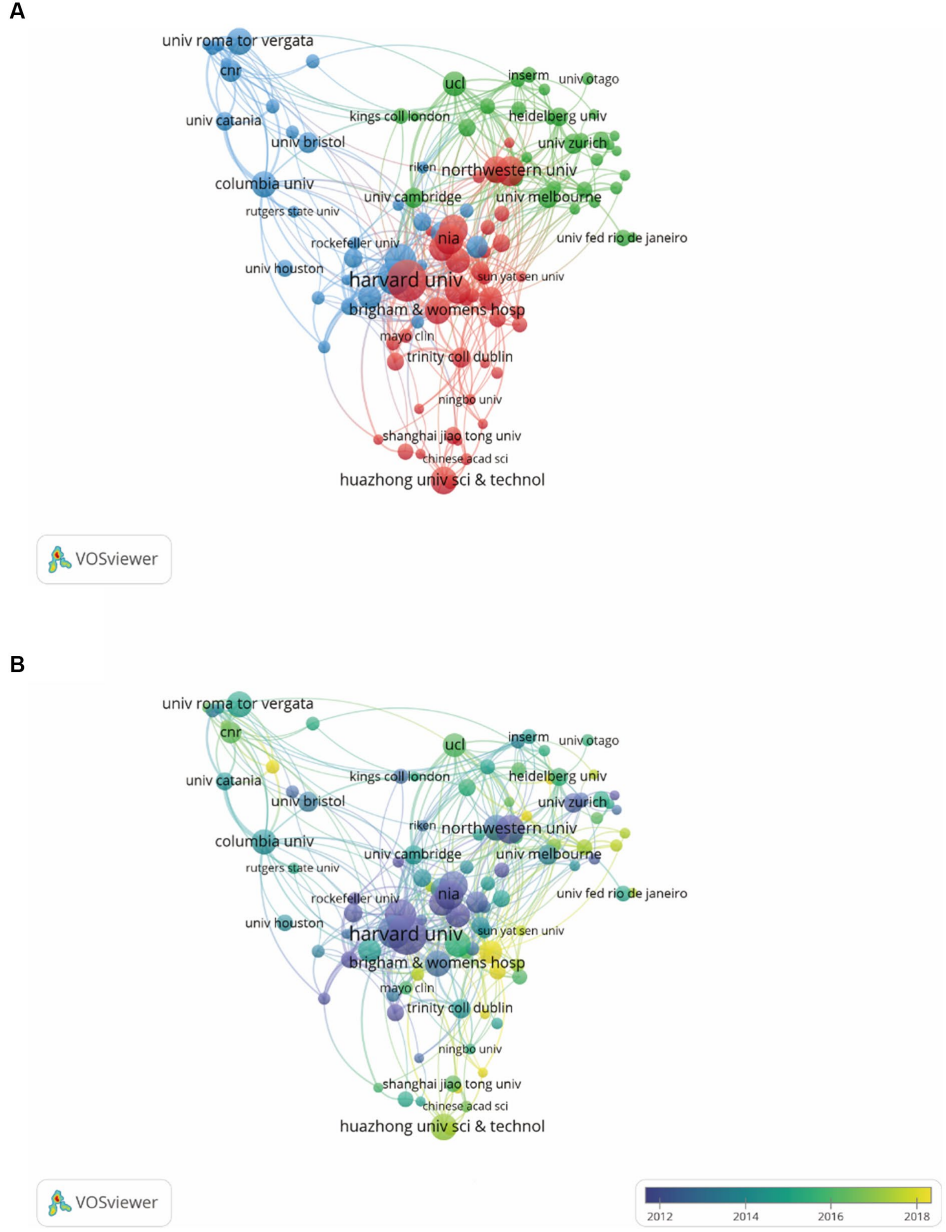
VOSviewer analysis of collaborations among different institutions. **(A)** The cooperation network map of institutions was based on VOSviewer. Size of the nodes represented the number of publications by each institution, and links between two nodes represented the frequency of the cooperation between two institutions. **(B)** The color of each circle referred to the average publication year for the institution.

In order to find out about the research institutions and interinstitutional cooperation efforts in the field of synaptic plasticity in AD, we conducted a visualization network map by VOSviewer (Figure 3A). The size of the circles represented the number of publications, therefore, the institution with more published articles tended to present larger circles. Links between the two institutions meat that they have collaboratively published articles. The length of the lines indicated the strength of the cooperation. The research network presented that Harvard University was the most productive institution and had the most frequent collaboration with other institutions. However, the cooperation among other institutions was relatively not close enough. As shown in Figure 3B, Harvard University, University Calif San Diego and National Institute of Aging had the most publications concentrated between 2012 and 2014, while publications from University Toronto, Columbia University and University College London were mainly published after 2016. Furthermore, Table 1 showed the top 10 prolific institutions. Seven institutions located in the United States, two located in China, and one located in Canada. Through analyzing these data, we can indicate that Harvard University contributed the most in the field of synaptic plasticity in AD followed by New York University and the University of San Diego.

**Table 1:** List of top 10 organizations.

Organization	Documents	Citations	Total link strength
*Harvard Univ*	64	10,301	414
*NYU*	49	4,618	229
*Univ Calif San Diego*	47	3,642	144
*NIA*	41	7,555	136
*Northwestern Univ*	41	6,498	265
*Univ Calif Irvine*	41	6,821	143
*Shanxi Med Univ*	39	1,162	46
*Huazhong Univ Sci & Technol*	38	1,064	40
*Univ Toronto*	37	1,749	89
*Brigham & Women’s Hosp*	36	4,728	277

### Bibliometric analysis of journals

**Figure 4:**
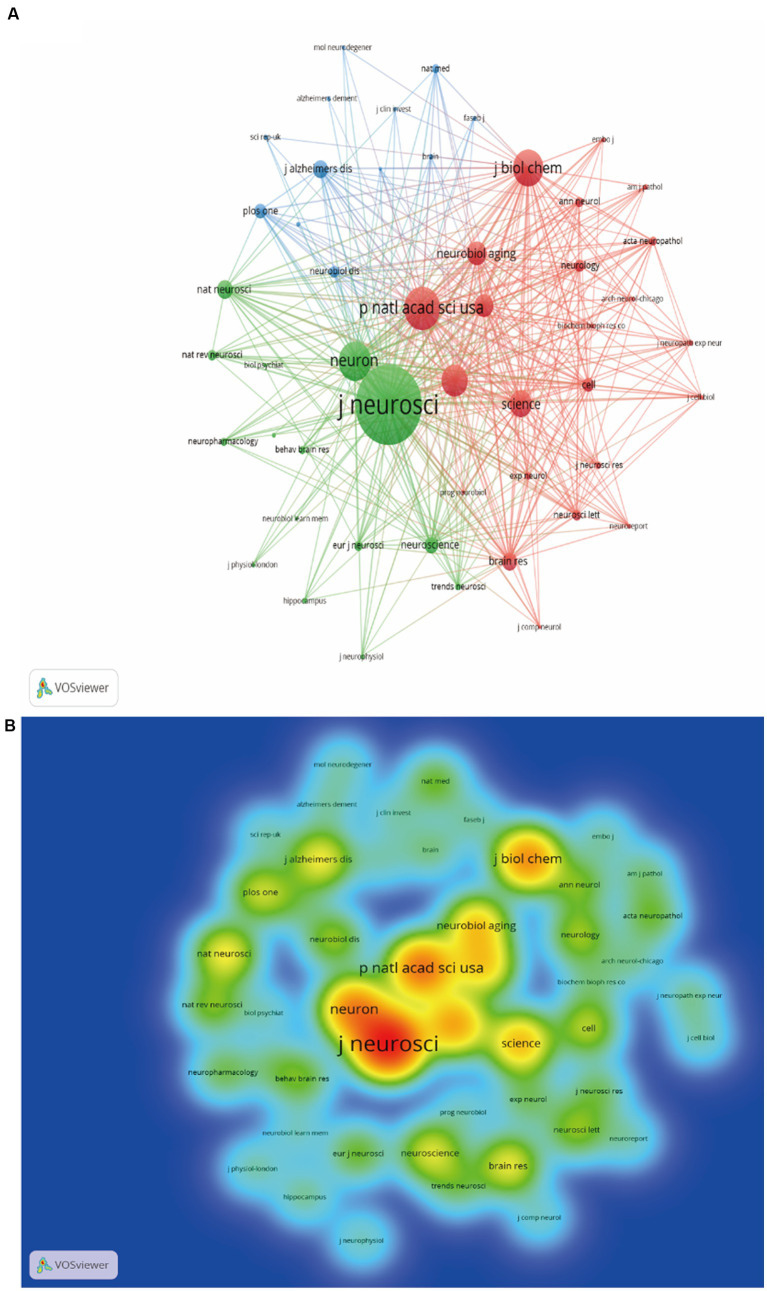
Analysis map of the most prolific journals. **(A)** The co-citation network visualization map of journals was based on VOSviewer. **(B)** The density visualization map of journals was based on VOSviewer.

The WoSCC search showed that a total of 235 journals participated in the publication of synaptic plasticity in AD. Table 2 listed the top 10 productive journals, and the most prolific one was the journal of Alzheimer’s disease which published 256 documents, followed by the Journal of Neuroscience (164) and Neurobiology of Aging (144). The co-citation network visualization map was shown in Figure 4A. The size of the node was correlated with the number of citations of each journal and this distribution trend could be understood more clearly in Figure 4B. The highest cited one was the journal of neuroscience with 17,166 citations, followed by Proceedings of the national academy of sciences of the United States of America-Physical sciences (9439) and Neuron (8549).

**Table 2:** List of top 10 journals.

Journals	Documents	Citations	Total link strength
*Journal of Alzheimer’s Disease*	256	8,114	778
*Journal of Neuroscience*	164	19,782	995
*Neurobiology of Aging*	144	6,822	499
*Journal of Neurochemistry*	90	5,955	336
*Molecular Neurobiology*	85	2,644	251
*Frontiers in Aging Neuroscience*	79	1,624	202
*Neurobiology of Disease*	72	4,324	337
*Neuroscience*	67	2,230	174
*Neuropharmacology*	65	3,433	262
*Behavioral Brain Research*	54	2,841	206

### Bibliometric analysis of authors

**Figure 5:**
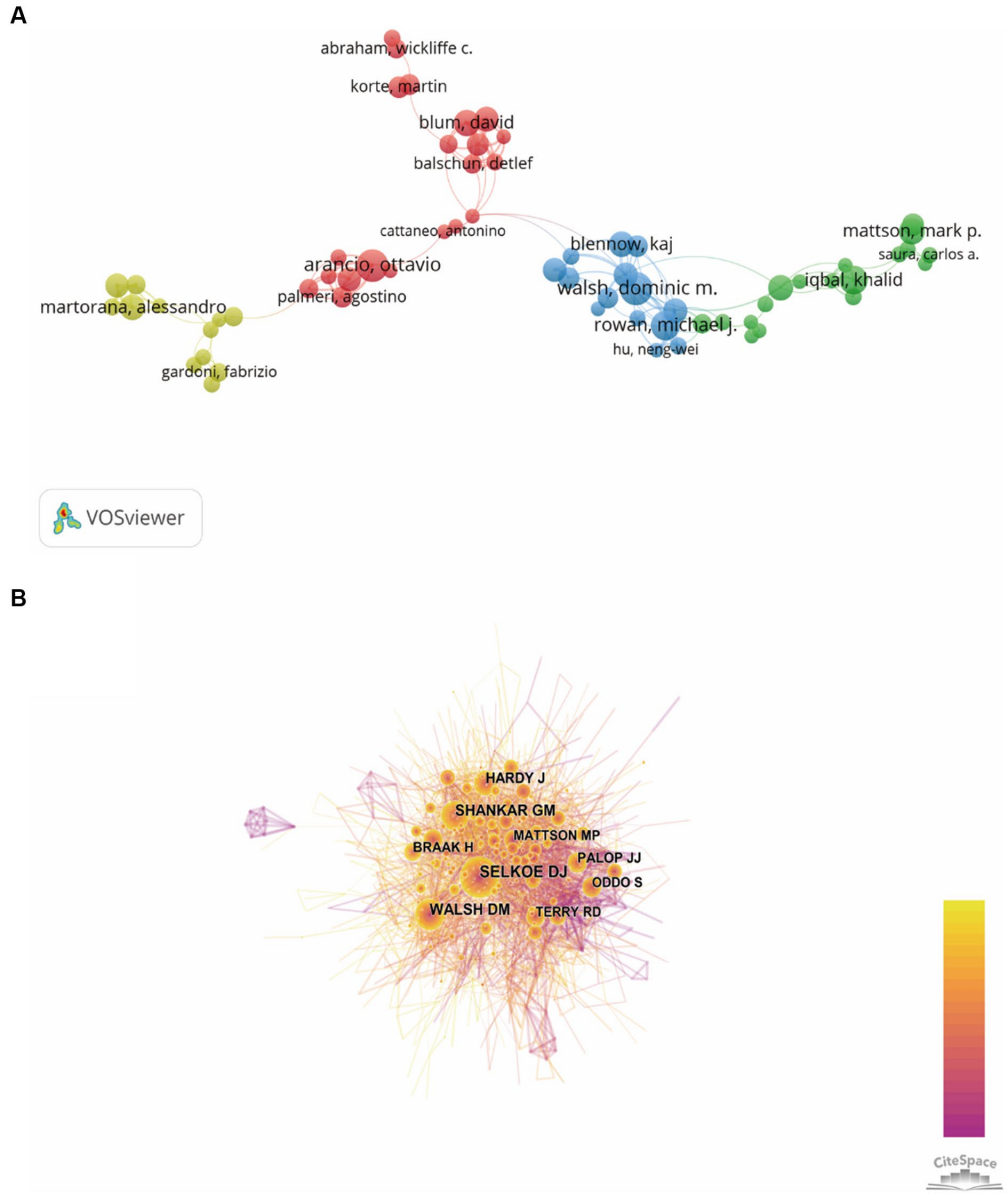
Visualization map of authors generated by VOSviewer and CiteSpace. **(A)** The co-authorship network visualization map of authors was based on VOSviewer. Circle size meat the number of authors’ publications, and the length of lines meat the frequency of collaboration between those two authors. **(B)** The co-citation network visualization map of authors was based on CiteSpace. The node size represented the number of citations of each author, while the links referred to the frequency of the co-citation between two authors.

The authors and their collaborations regarding synaptic plasticity in AD were shown in Figure 5A. In this visualization map, there were only four clusters, indicating that the collaboration of authors was not very close. The co-citation analysis of authors could reveal the core researchers and their contributions to a certain field. We conducted a visualization map of co-cited authors *via* CiteSpace (Figure 5B). Selkoe DJ from Harvard Medical School was the most cited author and has been cited 872 times in 2002. Shankar GM from Harvard Medical School has been cited 630 times in 2007 and thus ranks second, followed by Walsh DM (593), Hardy J (432) and Braak H (313). The other five major researchers and their institutions were also presented in Table 3 (Palop JJ from the University of California, Terry RD from the University of California-San Diego, Mattson MP from the Johns Hopkins University School of Medicine, Oddo S from the University of California, and Lambert MP from Perelman School of Medicine). It should be noted that the top three productive researchers called Selkoe DJ, Shankar GM and Walsh DM, were all members of Harvard Medical School. Consequently, it could be concluded that Harvard Medical School played a crucial role in this field.

**Table 3:** List of top 10 authors.

Author	Citations	Institution
*Selkoe DJ*	872	*Harvard Medical School*
*Shankar GM*	630	*Harvard Medical School*
*Walsh DM*	563	*Harvard Medical School*
*Hardy J*	432	*University College London*
*Braak H*	313	*J.W. Goethe University*
*Palop JJ*	312	*The University of California*
*Terry RD*	298	*The University of California-San Diego*
*Mattson MP*	295	*The Johns Hopkins University School of Medicine*
*Oddo S*	295	*The University of California*
*Lambert MP*	275	*Perelman School of Medicine*

### Bibliometric analysis of references

**Figure 6:**
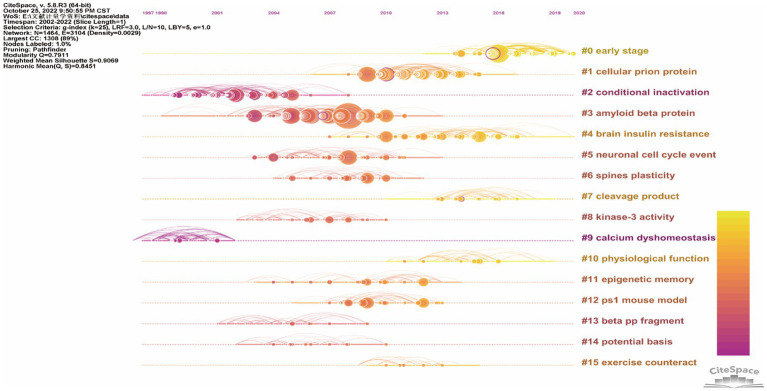
The timeline view of co-citation references clusters based on CiteSpace. The bold timeline indicated that the clustering topic was a hotspot during that period of time. Citation tree rings which had different sizes on the timeline represented some key documents with a high citation frequency.

In order to discover the present main topics and the evolution of synaptic plasticity in AD, the timeline view of co-citation documents clusters was conducted (Figure 6). It showed that in this scientific field, the focus of research seems to have shifted from conditional inactivation and calcium dyshomeostasis to early stage and brain insulin resistance. The top five highly cited references were presented in Table 4. Results showed that the highest cited reference was Amyloid-beta protein dimers isolated directly from Alzheimer’s brains impair synaptic plasticity and memory which was published by Nature Medicine in 2008. It suggested that soluble Aβ oligomers can effectively inhibit LTP, enhance LTD, reduce dendritic spine density and also disrupt the memory of learned behavior in normal rats. This research denoted that soluble Aβ oligomers extracted from the cerebral cortex of AD brains can potently impair synapse structure and function (Shankar et al., 2008), which was consistent with the results of the article with the second highest citation. Researchers demonstrated that without monomers and amyloid fibrils, Aβ oligomers can still disrupt synaptic plasticity *in vivo* (Walsh et al., 2002). The third highest-ranking article was published by Science in 2002, which suggested that synapses were the initial target in AD and its dysfunction appeared before Aβ plaque formation (Selkoe, 2002). This provided us with a new idea to intervene in the development of AD in the early stage. These high-citation references have made significant contributions in this field and deserve more attention in the future.

**Table 4:** List of top 5 high cited reference.

Reference	Total citations	Author (year)
*Amyloid-beta protein dimers isolated directly from Alzheimer’s brains impair synaptic plasticity and memory*	512	Shankar et al. (2008)
*Naturally secreted oligomers of amyloid β protein potently inhibit hippocampal long-term potentiation in vivo*	397	Walsh et al. (2002)
*Alzheimer’s Disease Is a Synaptic Failure*	367	Selkoe (2002)
*The amyloid hypothesis of Alzheimer’s disease: progress and problems on the road to therapeutics*	340	Hardy and Selkoe (2002)
*Diffusible, nonfibrillar ligands derived from Abeta1-42 are potent central nervous system neurotoxins*	265	Lambert (1998)

### Bibliometric analysis of co-occurring keywords and burst detection with keywords

**Figure 7:**
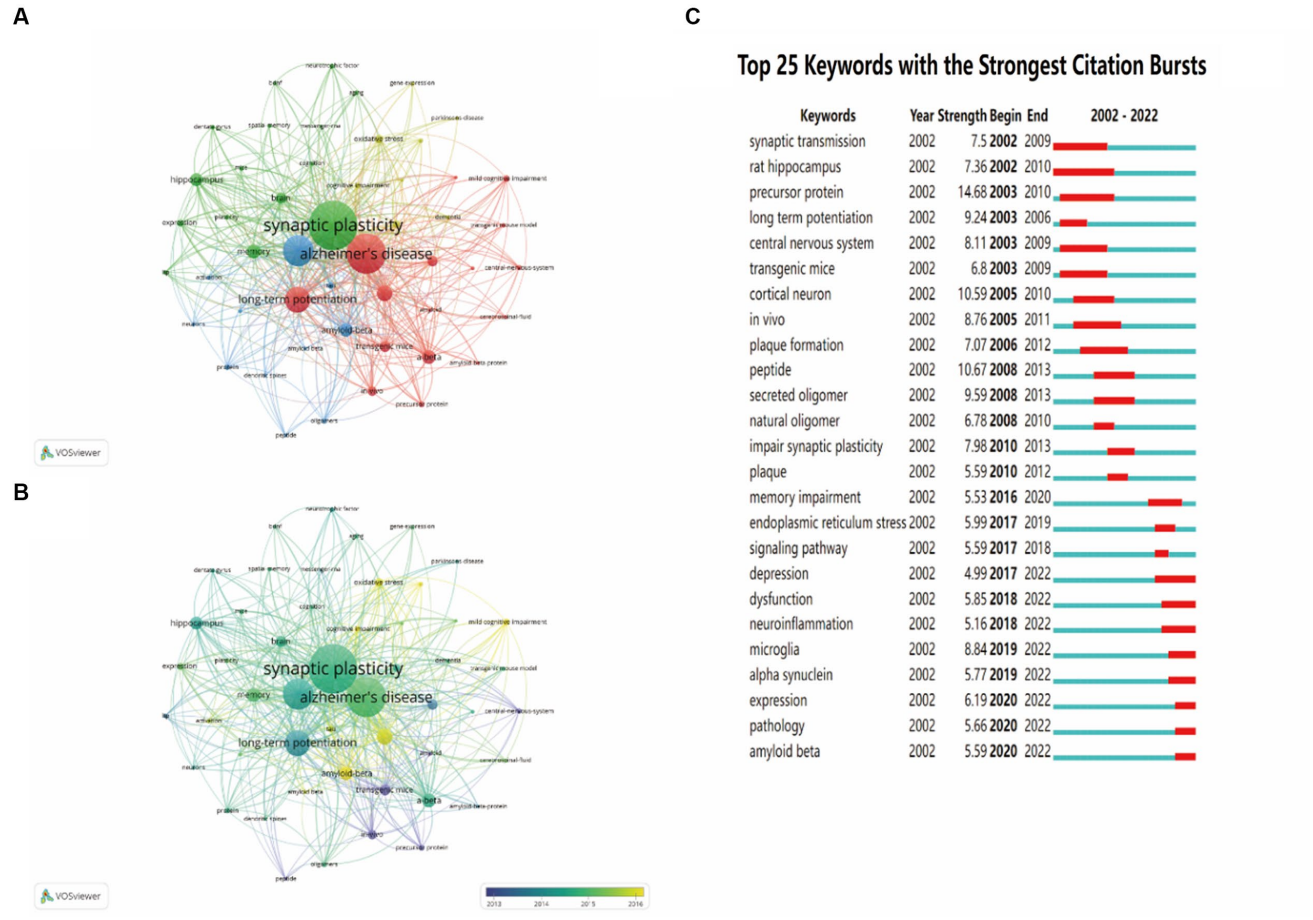
The analysis of keywords co-occurrence and keywords citation bursts. **(A)** The co-occurrence network visualization map of all keywords was based on VOSviewer. **(B)** The overlay visualization map of all keywords was based on VOSviewer. **(C)** The top 25 keywords with the strongest citation bursts were based on CiteSpace. Keywords marked in red indicated a sudden increase in usage frequency of this keyword during that period. Blue represented a relatively unpopular period of time.

To identify the development in the field of synaptic plasticity in AD, we presented 50 keywords with a minimal occurrence of 87 times and classified into four clusters in Figure 7A. The size of each circle was positively correlated with the occurrence frequency of keywords. The green cluster contained some keywords such as synaptic plasticity, memory, neurotrophic factor and dentate gyrus. The red cluster contained some keywords such as Alzheimer’s disease, long-term potentiation and amyloid precursor protein. In the yellow cluster, the main keywords were oxidative stress, dementia and neurodegeneration. The blue cluster keywords were dendritic spines, oligomers and phosphorylation. The keywords in the same cluster showed some correlation with each item. In Figure 7B, circles were colored differently according to the average occurrence time. It presented that “precursor protein,” “peptide” and “central nervous system” have been researched earlier than 2014, whereas keywords such as “oxidative stress” and “mild cognitive impairment” were the current research focus in this field and may become hotspots in the future. Keywords burst detection was conducted to identify the future emerging trends and current research hot topics. The top 25 keywords with the strongest citation burst were shown in Figure 7C. The red line indicated that the use of this keyword suddenly increased during this period of time and showed its beginning and ending years. In contrast, a blue line represented relative unpopularity. In the past two decades, precursor protein ranked first with the highest burst strength (14.68), followed by peptide (10.67), cortical neuron (10.59), secreted oligomer (9.59) and long-term potentiation (9.24). Some keywords burst with long durations, such as rat hippocampus, synaptic transmission and precursor protein, with a time span of 9, 8 and 8 years, respectively. In addition, the hot topics have switched from synaptic transmission, rat hippocampus, precursor protein and cortical neuron to plaque formation, secreted oligomer, impair synaptic plasticity and endoplasmic reticulum stress, and finally to depression, neuroinflammation, microglia, alpha synuclein and amyloid beta. This transition indicated that neuroinflammation, microglia and alpha synuclein have drawn the attention of peer investigators, indicating that they have become the new current research hotspots.

## Discussion

In this study, we conducted bibliometric analysis *via* CiteSpace and VOSviewer to visually analyze a total of 2,348 publications from 2002 to 2022. We aimed to reveal the current research hotspot of synaptic plasticity in AD intuitively and provide guidance for future studies.

### Research trends of synaptic plasticity in Alzheimer’s disease

From the bibliometric analysis on the role of synaptic plasticity in AD publications over the past two decades, it was found that the number of published articles has gradually increased, indicating that synaptic plasticity was generally attracting attention in this field. 52 countries and 51 institutions were analyzed, respectively, by online bibliometric platform and VOSviewer. According to the results (Figure 2B), the United States, China, Italy and Germany contributed most in this field. By analyzing the institutional publication numbers and citations among all institutions, we figured out that Harvard University was the most productive one (Table 1). Based on these data, the United States is undoubtedly the world leader in this field. Meanwhile, our analysis showed that the United States and China had the most frequent cooperation because there are close academic exchanges between researchers in these two countries. By contrast, connections between other countries and organizations remain weak. This indicated that we should pay more attention to the cooperation between organizations in different countries in order to promote the development of this field. In China, AD has become one of the most urgent neurodegenerative diseases that needs to be solved. Researchers continuously try to find a new therapy method and have published substantial articles. However, the number of publications and citations is still insufficient, which indicates Chinese researchers need to further improve the quality and influence of their publications.

In order to evaluate the contribution of authors in this field, we ranked them based on their total number of citations. According to the data extracted from WoSCC, we found that Selkoe DJ ranked first with the most number of citations, followed by Shankar GM and Walsh DM. It should be noticed that these top three authors all come from Harvard Medical School, which was the most prolific institution we have referred above. Moreover, we also conducted the co-authorship visualization map to provide researchers the current partnerships and confirm potential collaborators.

Analyzing popular journals can provide researchers with a definite searching direction in this scientific research domain. We finally found that the Journal of Alzheimer’s Disease has published the most documents, while the Journal of Neuroscience ranked first by citations (Table 2). In terms of publications and citations, we can conclude that the most influential one was the Journal of Neuroscience. Through the journals’ rank, investigators can quickly find suitable journals for their own publications.

Through analyzing the timeline view of related references and the keywords citation bursts, we can understand the hotspots, research fronts and the evolution of this scientific research field. At the early stage, the effects of synaptic transmission, precursor protein, plaque formation and secreted oligomer have been studied over 5 years which indicated that these factors attracted researchers’ attention and composed the research foundation in the field of synaptic plasticity in AD. Meanwhile, our research demonstrated that early stage, neuroinflammation, microglia and alpha synuclein have become the focal points of recent studies.

### Research focus of synaptic plasticity in Alzheimer’s disease

Publications with the highest citation number had been correlated with tremendous academic impact on a certain research field. Therefore, we analyzed the top 10 highly cited publications in recent 5 years, the results showed that the main influenced factors of synaptic plasticity in AD were therapeutic strategies, astrocyte and amyloid-β protein.

At present, despite comprehensive research into the pathophysiology of AD and a mass of drugs entering clinical development, no effective new drug has been approved since memantine in 2003 (Panza et al., 2019). Many reasons have been put forward to explain this failure, such as inappropriate patient selection, suboptimal dosing, drug exposure (‘too little, too late’) and inappropriate time of intervention (Polanco et al., 2018). Furthermore, the lack of a detailed understanding of the AD pathophysiology might lead to select of the wrong targets. Most of the drugs for AD target the accumulation of Aβ peptide. For example, AN-1792, CAD106, solanezumab and gantenerumab can stimulate Aβ clearance, while verubecestat, lanabecestat and elenbecestat can decrease Aβ production (Panza et al., 2019). Notably, anti-diabetes drugs, intervention of neuroinflammation and tau-targeting therapies have become promising new targets for treating AD. Craft et al. demonstrated that intranasal insulin detemir or regular insulin can effectively improve cognition and daily functioning of AD patients (Craft et al., 2017). In the early years, researchers found that patients given some anti-inflammatory drugs could reduce the risk of developing AD (Abbott, 2018). Microglia gather around amyloid plaques and the areas of brain degeneration. Many studies have revealed that microglia have a central role in the link between inflammation and neurodegeneration (Zhou et al., 2020). Initially, the moderate activation of microglia can surround plaques and degrade Aβ by phagocytosis. Nevertheless, chronic overactivation of microglia tend to act a more proinflammatory role and less phagocytic ability (Marsh et al., 2016). It is necessary to fully understand the biology of microglia to consider how to design immune-based therapies for AD. Another promising target for intervention is tau. Although most of the anti-tau therapies have failed because of toxicity and/or lack of efficacy, the current new tau-targeting therapies have shown prospective effect in numerous preclinical studies (Mummery et al., 2023).

In recent years, the role of astrocytes in AD has also received increasing attention. It has been reported that astrocytes play an important role in regulating structural remodeling and functional plasticity of synapses (Santello et al., 2019). In addition, dynamic cellular imaging work revealed that astrocytes can intimately interact with neurons, while astrocyte-targeted mouse genetics illustrated that astrocytes participate in memory processes (Bindocci et al., 2017; Stobart et al., 2018). Despite the technical advances helping researchers further understand the role of astrocytes, they also highlight the incomplete comprehension of astrocyte biology. In pathological conditions, such as AD, astrocyte-neuron interactions can be substantially disrupted, with strong effect on brain circuits supporting memory formation and cognitive function (Santello et al., 2019). These findings suggest that therapeutic strategies targeting astrocyte pathways may have tremendous potential to fight against cognitive deficits in AD.

Walsh et al. revealed the influence of soluble Aβ oligomers on synaptic plasticity and memory (Walsh et al., 2002). They found that these soluble Aβ oligomers potently inhibited LTP, enhanced LTD, reduced dendritic spine density and also disrupted the memory of learned behavior in normal rodents (Walsh et al., 2002). In addition, Aβ can bind to distinct components of neuronal and non-neuronal plasma membranes to induce complex patterns of synaptic dysfunction and network disorganization (Mucke and Selkoe, 2012). In contrast, insoluble amyloid plaque cores from the AD cortex did not significantly alter synaptic plasticity and LTP, suggesting that plaque cores are largely inactive. However, this does not mean that insoluble amyloid plaque cores have no pathogenic role. Their accumulation may indicate that they act as reservoirs of small bioactive oligomers, and may release locally active Aβ species *in vivo* (Hardy and Selkoe, 2002; Yang et al., 2017). Hence, these studies concluded that soluble Aβ oligomers can impair synapse structure and function, and dimers are the smallest synaptotoxic species (Shankar et al., 2008). In addition, Aβ oligomers can interact with astrocytes and microglia. For instance, Aβ oligomers have been demonstrated to trigger astrogliosis and induce ROS generation in activated astrocytes (Wyssenbach et al., 2016; Pereira Diniz et al., 2017). As for microglia, it is possible that Aβ oligomers have a role in attracting microglia to plaques, while Aβ oligomers can also trigger a switch in microglial phenotype to pro-inflammatory phenotype, leading to produce excessive inflammatory factors (El-Shimy et al., 2015; Cline et al., 2018). These inflammatory factors, such as aberrant tumor necrosis factor (TNF), can cause synaptic dysfunction and memory loss (Ledo et al., 2016). Thus, the role of Aβ oligomers in the pathogenesis of AD is indispensable.

### Research fronts of synaptic plasticity in Alzheimer’s disease

Research fronts are the emerging hotspots or research topics. The timeline view of co-citation references clusters revealed that “early stage” has increasingly gained researchers’ attention. Neuroinflammation, microglia and alpha synuclein have become the focal points of recent studies according to the analysis of keywords citation bursts. Although the hypothesis of amyloid-β deposition and neurofibrillary tangles can explain many aspects of AD pathogenesis, there still exist many pathological processes that cannot be sufficiently illustrated with this hypothesis. Hence, in the following discussion, we particularly focused on the emerging topic terms and the associated publications, which can provide researchers new insights into the field of AD.

Neuroinflammation plays a significant role in the pathogenesis of AD with the discovery of increased levels of inflammatory markers. In addition, AD risk genes are identified associated with innate immune functions in patients. Neuroinflammation is intended to be a defense mechanism that can protect the body from removing or inhibiting diverse pathogens, but sustained inflammatory responses can induce neurotoxicity (Hickman et al., 2018).

In recent years, many researchers are prone to investigate the role of microglia in the development of AD. According to the results of keywords citation bursts, microglia has become one of the research hotspots. Microglia is the resident macrophage of the central nervous system (CNS) and the first responder to pathological insults, which play a critical role in regulating inflammatory processes of the CNS (Morales et al., 2014; Unger et al., 2020). It can act as the antigen-presenting cell to phagocytose the toxic product and release cytotoxic factors in order to protect the brain (Morales et al., 2014). Under normal conditions, microglia primarily exist in a resting state and have significant physiological functions in the regulation of synaptic transmission, neuronal activity and synaptic pruning (Akiyoshi et al., 2018). However, microglial excessive activation or dysfunction is positively correlated with cognitive impairment (Cornell et al., 2021). As for AD patients, extracellular amyloid-β and/or intraneuronal phosphorylated tau can both activate microglia (Merighi et al., 2022). Recent studies have proved that microglia participate in the modulation of synaptic plasticity, including LTP and LTD which are the cellular mechanism of learning and memory (Innes et al., 2019; Zhou et al., 2019). In the healthy brain, there is a balance between proinflammatory and anti-inflammatory factors released by microglia, while if disrupted, it can influence synaptic plasticity (Golia et al., 2019). Traditionally, microglia can be divided into the M1 (classically activated) and M2 (alternatively activated) based on their activation pattern. However, the switch between two definite phenotypes of microglia and astrocytes is complicated and may differ with the severity and stage of neurodegenerative disease (Kwon and Koh, 2020). At the beginning of Aβ pathology, microglia can exert a neuroprotective role by degrading and removing Aβ and tau, while with the evolution of AD, increasing in the size and number of amyloid plaques, the clearance ability of microglia decrease (Mawuenyega et al., 2010). Different species of Aβ aggregate can induce microglia activation and the production of pro-inflammatory cytokines (such as IL-1β, IL-6, IL-8 and TNF) in order to exhibit the neuroprotective effect, but these microglia cells later transfer to the neurotoxic (pro-inflammatory) phenotype resulting in neuronal dysfunction and death (Jimenez et al., 2008; Tang and Le, 2016). Pettigrew et al. reported that overexpression of TNF-α results in an increase in LTP, suggesting the synaptic networks may be hyperexcitable (Pettigrew et al., 2016). Nevertheless, compared to TNF-α, IL-β, another proinflammatory factor released by microglia, impairs LTP in the CA1 region (Hoshino et al., 2017). In conclusion, these results indicate that proinflammatory cytokines released by microglia have diverse effects on LTP and synaptic plasticity.

In addition to microglia, T cells, the crucial immune cells of the adaptive immune system, are also involved in AD pathology (Dai and Shen, 2021). During AD progression, activated CD8+ T and CD4+ T cells, the two major T-cell subsets, can gradually infiltrate into brain parenchyma due to increased permeability of the blood–brain barrier (BBB) (Cheng et al., 2014). Increased T cells promote crosstalk with microglia in the brain. Depletion of microglia eliminates T cell infiltration and depletion of T cells also largely blocks microglia activation (Chen et al., 2023). Recent studies demonstrated that the number of T cells were increased in brain regions with tauopathy rather than amyloid pathology alone (Merlini et al., 2018; Chen et al., 2023). Depleting T cells significantly reduce p-tau staining and tau-mediated neurodegeneration, demonstrating the critical role of T cells in mediating tau pathology (Chen et al., 2023). Further illuminating the correlation between T cells and tau protein might yield novel therapeutic targets for preventing neurodegeneration in AD.

Alpha-synuclein (α-syn) is considered the primary constituent protein of Lewy bodies, which is the defining hallmark of Lewy body dementia (LBD) including Parkinson’s disease dementia (PDD) and dementia with Lewy bodies (DLB) (Hijaz and Volpicelli-Daley, 2020; Koga et al., 2021). α-Syn located at pre-synaptic terminals and was correlated with the distal reserve pool of synaptic vesicles. Knocking down or overexpression of α-syn resulted in deficiencies in synaptic transmissions, demonstrating that α-syn played a significant role in the regulation of neurotransmitter release and synaptic plasticity (Lashuel et al., 2013). α-Syn oligomers rather than monomers or fibrils can impair LTP through activated NMDA receptor (Diógenes et al., 2012). Consequently, the synaptic plasticity was compromised in AD patients. In recent years, researchers confirmed that α-syn oligomers could promote the formations of Aβ oligomers and stabilize their cross-β structures, whereas α-syn monomers suppressed Aβ aggregation, indicating that distinct structural forms of α-syn had different influences on Aβ aggregation (Atsmon-Raz and Miller, 2016; Chia et al., 2017; Shim et al., 2022). Hence, subsequent studies should be more focused on the different structural forms of α-syn to further illustrate the correlation between α-syn and Aβ. In addition, α-syn has also been reported as a protein that can directly interact with tau (Pan et al., 2022). The two proteins could promote mutual homogenous/heterogeneous aggregations. For example, α-syn could induce tau aggregation, in turn, tau could facilitate the fibrillization of α-syn (Hijaz and Volpicelli-Daley, 2020). Previous studies have demonstrated that the C-terminal of α-syn can directly interact with the microtubule binding domain of tau (Waxman and Giasson, 2011; Dasari et al., 2019). Interactions between these two proteins also accelerated tau phosphorylation, suggesting that increased α-syn in AD may promote tau pathology and further exacerbate cognitive decline (Bassil et al., 2020; Hu et al., 2020). Altogether, α-syn could be an emerging pathophysiological biomarker of AD and it may be crucial for assessing potential therapeutics for AD.

### Limitations

The current study had several limitations. First, we only analyzed two document types (reviews and articles), other publications such as books or conference papers are not collected by bibliometric searches. Second, only English publications were enrolled in our research. Other significant articles using non-English languages, although few, were not considered. Third, we only indexed the documents in the WoSCC database due to the requirements of the CiteSpace software. Therefore, we may not analyze relevant articles only found in other databases such as PubMed and Scopus.

## Conclusion

This study provided knowledge about synaptic plasticity in AD from a visualization and bibliometric perspective. We analyzed the publication trends hotspots and research fronts. In terms of publication trends, research on synaptic plasticity in AD steadily increased. Currently, the hotspots and research fronts have transferred from the synaptic transmission and precursor protein to neuroinflammation, microglia and alpha synuclein. Apart from the mechanism of Aβ peptides and hyper phosphorylated tau, researchers attempt to discover new mechanisms to explain some pathological processes that cannot be well illustrated with the traditional mechanism. This bibliometric study is beneficial for researchers to understand AD better by analyzing these development trends.

## Data availability statement

The raw data supporting the conclusions of this article will be made available by the authors, without undue reservation.

## Author contributions

JY conceived the project and designed the studies. YZ and JZ searched the literature together. YZ and YW analyzed the data. JY and YZ drafted the figures and wrote the manuscript. All authors contributed to the article and approved the submitted version.

## Funding

This study was supported by grants (No. 82171183 and No. 81771269) from the National Natural Science Foundation of China. The funders had no role in the study design, data collection and analysis, decision to publish, or preparation of the manuscript.

## Conflict of interest

The authors declare that the research was conducted in the absence of any commercial or financial relationships that could be construed as a potential conflict of interest.

## Publisher’s note

All claims expressed in this article are solely those of the authors and do not necessarily represent those of their affiliated organizations, or those of the publisher, the editors and the reviewers. Any product that may be evaluated in this article, or claim that may be made by its manufacturer, is not guaranteed or endorsed by the publisher.
